# Heart-Lesions in Relation to Mental Symptoms
*Read at a meeting of the Dorset and West Hants Branch of the British Medical Association.


**Published:** 1886-09

**Authors:** P. Wm. MacDonald

**Affiliations:** Senior Assistant Medical Officer, Dorset County Asylum


### HEART-LESIONS IN RELATION TO MENTAL SYMPTOMS.*

BY

P. WM. MACDONALD, M.D.,

*Senior Assistant Medical Officer, Dorset County Asylum.*

---

Notwithstanding that the ancients were wont to class insanity as a bodily disease,—for Hippocrates taught that mental diseases were due to the circulation of black bile in the blood,—our present knowledge of the physical causation of mental disease is sadly deficient and most imperfect.

Within a comparatively short lapse of time, I have been fortunate enough to meet with a series of cases, in many of which the close sympathetic connection between mind and body was impressively and forcibly illustrated, and where, from the first, the symptoms were of a bodily-mental nature.

I had thus placed at my convenience, for study, a valuable collection of material, both clinical and pathological, and I trust that I have not wholly failed to take advantage of the same.

The subject of heart-lesions, viewed in relation to mental disease, is far too wide to be even summarised in the time at my disposal, and I shall confine my remarks to the part played by mental disease, with or without hypertrophy, in the production of mental symptoms, illus-

* Read at a meeting of the Dorset and West Hants Branch of the British Medical Association.

trating what I have to say by a specimen, and short notes of the case that produced it.

This case was an ordinary example of hypochondriacal melancholia, occurring, without any apparent cause, in a middle-aged man, who had previously enjoyed good health. The certificate on which he was admitted stated that the patient was restless, had delusions, and was suffering from brain-softening. On admission I noted that the rhythm of the heart was irregular and weak, and the mitral sounds muffled. As regards the mental state, he was low, depressed, miserable, and unhappy, yet could talk rationally. He fancied (at times) that he was ruined, and had ruined his family and home. He was reticent, and carefully evaded any question intended to elicit information of the past. Without being too brief, I may state that the mental symptoms did not change to any noticeable degree from the day of his admission till his death.

Eight months after admission he had a sudden attack of cardiac dyspnœa. The entire body was bathed in a cold clammy perspiration. The lips and extremities were livid; the pulse over 100, small and irregular; and he breathed as if each respiration would be his last. The dyspnœa was panting and gasping in its character, and very characteristic of mitral disease with pulmonary congestion. In the treatment of the case (*i.e.*, this attack) nitrite of amyl was prescribed, and with the best results; for in less than ten minutes the pulse had become stronger, more regular, and numbered 80, and the patient was breathing quietly. Next day I made a careful examination of the heart and other organs, and, with one exception—the heart,—they were all in fair working order. It I found to be hypertrophied and dilated, with a loud mitral regurgitant bruit. After this the mental symptoms were more intense. During the next four weeks he had three similar attacks of cardiac dyspnœa, and died suddenly on the 29th of December, 1885—eight months and two weeks after admission, and four weeks after the first severe attack of cardiac failure.

At the autopsy, sixteen hours after death, the brain was found free from disease, as far as the naked eye could discern; and under the miscroscope, no definite pathological changes were observed. The heart, after being washed and emptied of all clots, weighed 30 oz. The aortic, tricuspid, and pulmonary valves were found competent. The mitral valve did not close. The left ventricle was hypertrophied and dilated, and all the other chambers more or less dilated. The specimen (which was shown at the meeting) explains itself better than words of mine can; and if we can place any confidence in statistics, I believe there are only a few heavier specimens on record.

Such are the crude, brief, and disjointed facts of an interesting case; and the question now is, Can we connect the heart-lesions with the mental symptoms?

Six months before the patient came under our notice, he was in the full enjoyment of ordinary good health. About this time his wife observed that her husband was unusually quiet, and did not take the same interest in his home and little garden. As weeks rolled by the symptoms deepened into regular "depression of spirits," and the doctor was called in. After several months of ineffectual treatment, with "consultations," the patient's removal to an asylum was recommended, and in due course we admitted him.

Here then was a man, insidiously, and without any apparent cause, undergoing a gradual increasing change of disposition; and, despite all the efforts of medical ingenuity, he got more and more estranged, until his best and dearest friends became his greatest enemies, and his mind the home of all that was miserable.

For some time we looked upon the case as a possible recovery; but when the true nature, *i.e.* the cause of the mental symptoms, was known, all hope of recovery vanished. At no time of his residence in the Asylum did he show symptoms which pointed to coarse brain-disease; and had we recognised earlier the grave nature of the heart-lesions, we should not have held out the hope of even a possible recovery.

"We know how acutely sensitive are the nervous centres and their peripheral arrangements of any departure from the normal." Such a condition of things, therefore, as that resulting from mitral regurgitation, with hypertrophy and dilatation, may lead to material interference with, and perversion of, their functions.

The due fulfilment of the normal functions of each individual organ calls for an adequate supply of nourishment, and specially is this a truism in the case of the brain.

As to the cause of the enormous hypertrophy, I am doubtful, but most probably it was due to a combination of nervous and nutritive causes; and from facts already mentioned, we know that it was not the result of obstructive valvular disease.

A hypertrophied heart is often an useful one, and as long as the hypertrophy in the present case was compensatory, all went well; but as soon as the compensatory nature of the hypertrophy failed, so soon had we interference with, and deviation from, the normal functions of distant organs, more especially the brain.

With this compensatory failure, we had dilatation of the left ventricle, also of the mitral valve, producing the insufficiency.

And here begin our mental troubles. When the heart failed to send to the brain a regular, sufficient, and well-oxygenated supply of blood, that great and mysterious central clock showed signs of weakness in the form of mental depression and distrust. I believe that the mental symptoms were due to incurable disease in a distant organ; viz., the heart. Is it then to be wondered that, with permanent and irremovable heart-lesions as the sole factors and causes of the mental aberration, recovery was unattainable, and death but too certain?

We may accept it as a general, though not a constant rule, that mitral disease produces symptoms of melancholia, and aortic lesions more frequently those of a maniacal type.

Many cases, illustrative of both types, are annually admitted into our asylums, and it behoves everyone who is likely to be called upon to give his opinion, either verbally or in writing, as to a patient's mental stability, to bear in mind this distant nature of the actual cause.

When we meet with a patient who is the subject of mental perversion, in whatever form, let us not be deluded or led astray by the prevalent and erroneous idea, that the case is necessarily one of coarse brain-disease. At the outset our minds ought to walk clear of pathological changes in the brain, and should be directed to the great bodily organs which are so intimately connected with the encephalon.

When we have satisfied ourselves that no disease exists, or that disease in other organs cannot be connected with the mental symptoms, then, but not till then, are we justified in attributing those symptoms to actual and grave pathological changes in the brain.

"Brain-softening" is a much-abused expression, and, like heart-disease, covers a multitude of sins.

In thus speaking of mental symptoms, I do not wish to be understood to include cases where paralysis or other symptoms indicative of gross brain-lesions exist.

Let me urge upon you the importance, nay more, the absolute necessity, of detecting, diagnosing, and treating, at the earliest possible date, the true cause of the premonitory symptoms of mental disease.

Just as a single spark of fire, extinguishable if at once discovered, may cause a conflagration of many hours' duration; so may a small and trivial lesion, removable by early and judicious treatment, cause the loss of a gifted and talented mind.

